# Cholera Precautions

**Published:** 1893-07

**Authors:** 


ARTICLE VIII.

### CHOLERA PRECAUTIONS.

From a long list of wise recommendations given in the *Therapeutic Gazette* we glean the following:

Keep your presence of mind in the danger; avoid too great anxiety, for it clouds your clear judgment. Only the man who thinks clearly can make proper use of the precautions against danger.

Maintain cleanliness of your person and surroundings. Discretion, temperance, precise cleanliness, prove the best protection against disease.

Hold firmly to your ordinary regular mode of life Avoid festivities and assemblages of people.

Do not leave your home in order to escape the disease. Consider that you may be in greater danger in traveling, and living under altered conditions in a strange place, than while leading a careful, regulated life at home.

To make boiled water taste well, add to each glass (half a pint) as much tartaric acid as you can take on a knife-point, or two drops of hydrochloric acid.

Eat and drink nothing which is in a sick-room. Consider that flies and such insects might carry the germs of disease from the patient to your food.

Excretions of persons ill with, or suspected of having cholera, and the floor, etc , soiled with them, disinfect by copious (at least hourly) use of slack lime or chlorinated lime solution (5 drachms chlorinated lime to 1 quart cold water), or other trusted disinfectants.

If your digestion is disturbed, if you have diarrhœa, especially with vomiting or great nausea, consult a physician at once.

Until he comes, take a warm drink, put on a woolen bandage about your body, remain in your room; if in great distress, go to bed.

For relief, you may take a cup of tea, with cognac or rum. Let your food be a mucilaginous soup, also zwieback, or stale white bread without butter.

If you have reliable (prescribed from a physician's prescription) cholera drops at hand, take a dose.

Keep your presence of mind, even if you are ill. Fright and cowardice act unfavorably on body and mind. —*Md. Med. Journal.*

